# Lagrangian-like Volume Tracking Paradigm for Mass, Momentum and Energy of Nearshore Tsunamis and Damping Mechanism

**DOI:** 10.1038/s41598-018-32439-6

**Published:** 2018-09-21

**Authors:** Dae-Hong Kim, Sangyoung Son

**Affiliations:** 1 Department of Civil Engineering, University of Seoul, Seoul, South Korea; 2 School of Civil, Environmental and Architectural Engineering, Korea University, Seoul, South Korea

## Abstract

There is a gap between model- or theory-based research outputs, which suggest that the runup and amplification of nonbreaking waves generally increase as the sea bottom slopes decrease, and field observations, which indicate that tsunami damage has been rarely reported in places with vast continental shelfs. To resolve this contradiction, we propose a Lagrangian-like volume tracking paradigm to describe the energy, mass, and momentum of travelling nearshore tsunamis and apply the paradigm to analyse the tsunami damping mechanism at typical geophysical scales. The results support the following conclusions: (i) The suggested paradigm is consistent with field observations; continental shelfs with long and mild slopes can effectively diminish tsunami impacts. (ii) Potential energy becomes significant due to the energy transformation process on steeply sloped bathymetries. (iii) On mild-sloped bathymetries, tsunami potential and kinetic energies are conserved until breaking occurs. After breaking, undular bores attenuate tsunami energies effectively. (iv) For extended continental shelf bathymetries, more of the tsunami mass is reflected offshore.

## Introduction

For decades, various aspects of tsunami propagation have been studied, and the wave, bottom geometry, friction and wave-breaking characteristics have been regarded as the main factors of tsunami evolution. As inundation by tsunami is directly related to water surface elevation, interests have been mainly focused on the water surface elevation of tsunamis. From many analytical, experimental, and numerical studies, it was found that the runup height and amplification of nonbreaking waves on plane-like beaches generally increased as the bottom slope decreased^1–5^. However, field surveys show that tsunami damage has been rarely reported where vast continental shelfs with extremely gentle slopes exist, such as along the northern coasts of Australia, eastern coasts of China and western coasts of Korea. In contrast, certain part of the eastern coasts of Japan, India and Sri Lanka, where the seafloors are relatively steep, have experienced severe damage during tsunami attacks. Recently, Madsen *et al*.^6^ investigated these contradictory results; they examined the importance of the geophysical scale in tsunami studies and found that waves could evolve unrealistically without proper scale consideration.

Since tsunamis propagate over very long distances, bottom friction may play a significant role in tsunami attenuation. However, geophysical scale modelling results for tsunamis crossing oceans and continental shelfs has revealed that frictional dissipation was not primarily responsible for tsunami attenuation^7^. Zhao *et al*.^8^ applied a one-dimensional inviscid Boussinesq-type model to study these processes in the eastern sea of China and observed that the elevation of the main wave was lower where the shelf was long and mild. Since they used an inviscid model, the result implies that tsunami-damping resulted not only from the frictional effect but also from the shelf geometry. Therefore, it is difficult to conclude that bottom friction is one of the main drivers dissipating tsunami energy before tsunami runup occurs.

Wave breaking dissipation also has long been regarded as a non-negligible damping factor, especially when a main tsunami wave breaks. However, Madsen *et al*.^6^ reported that breaking did not occur in the main tsunami wave based on their tested conditions and 2004 Indian Ocean tsunami cases. Field observations show that wave breaking occurred in the short-crested undular bores riding on the top of the main tsunami wave. In addition, the breaking of bores over a relatively short distance made little impact on tsunami attenuation. However, although it seems reasonable to limit the contribution of the (main) wave breaking in the damping processes, we also need to be careful not to neglect the contribution of secondary wave breaking to damping in locations with very long continental shelfs.

**Figure 1:**
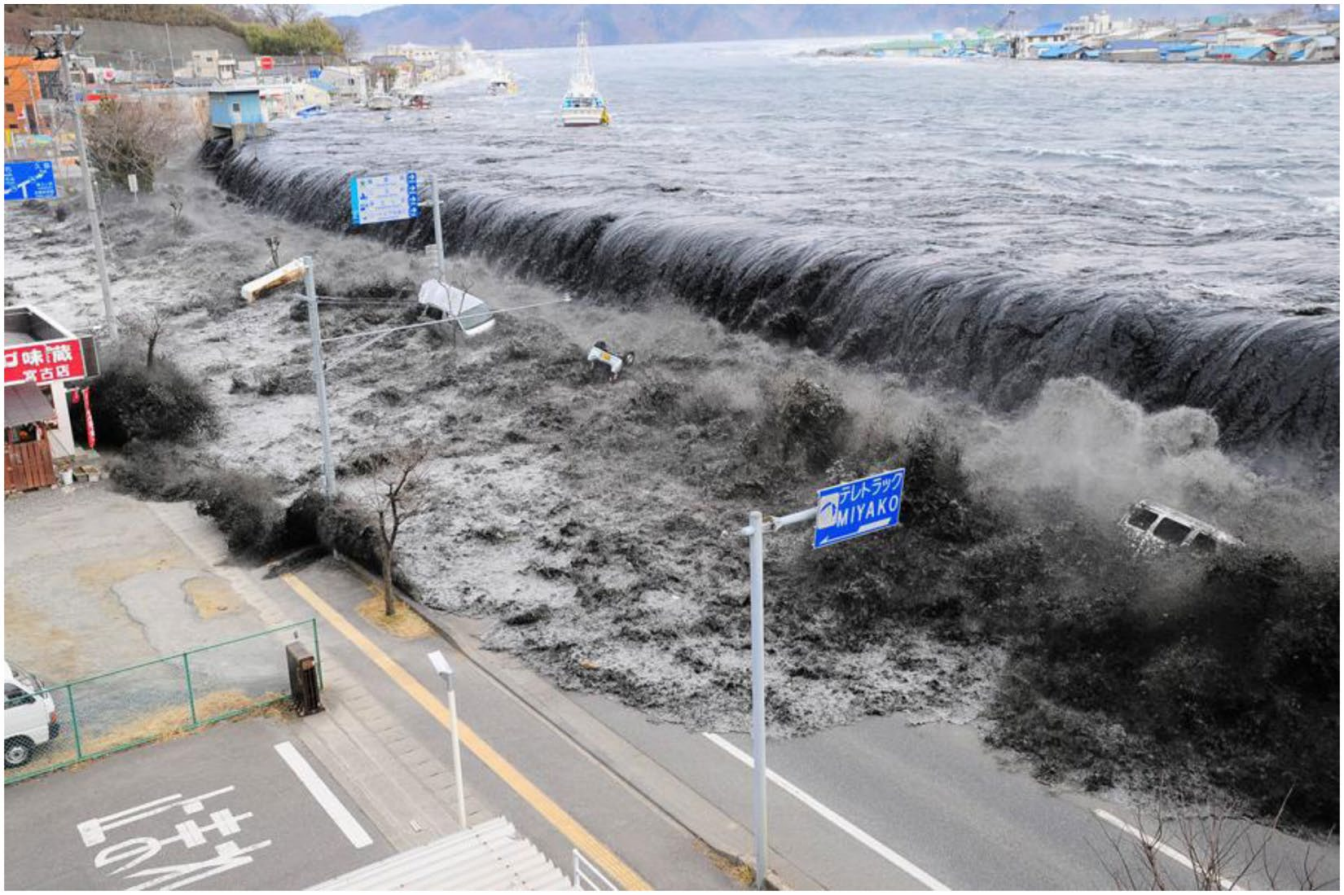
Tsunami overtopping a levee in Miyako City, Japan during the 2011 East Japan earthquake^26^.

Tsunami-induced inundation processes typically occur when the sea level is only a few feet higher than the crest of a levee, but a significant volume of water is discharged inland, causing catastrophic damage. For instance, Fig. 1 shows the moment when the tsunami overtopped the levee in Miyako City, Japan during the 2011 Japanese tsunami. Along with this observation, it is presumed that the water volume elevated above the levee crest was distributed over a very vast area and that the horizontal momentum towards the inland (or kinetic energy) was large enough to transport the elevated water inland. Based on this presumption, it is obvious that not only the water surface elevation but also the energy, volume, and momentum are important in assessing tsunami impacts. However, only a few previous studies have been based on the analysis of tsunami energy^9,10^.

Considering the abovementioned contradictions, the following questions arise: (1) Why is there a gap between model-, laboratory-, or theory-based research outputs and field observations? (2) How does a tsunami attenuate or amplify its physical properties at a geophysical scale (or, how should the travelling tsunami paradigm be described)? Hence, in this study, we present a detailed quantitative analysis on the energy, mass, and momentum paradigm of a travelling tsunami by following the moving tsunami volume with a Lagrangian-like frame rather than observing it as it passes through a fixed point. The results are used to provide a physical explanation underlying the linkage between seabed geometry and tsunami evolution and to examine evolution of the energy, mass, and momentum of a travelling tsunami at a typical geophysical scale. However, the proposed perspective on volume-tracking quantities is not implemented tangibly under the observational framework in the field because we cannot measure the height or flow velocity of the wave volume. Moreover, due to a very small vertical-horizontal aspect ratio, it is not well-suited for an experimental approach^6^; thus, this study is purely based on numerical simulations using a fully nonlinear, weakly dispersive, rotational and turbulent flow model. Although there are many additional factors, such as refraction and diffraction, influencing tsunami evolution, we limit the scope of this study to the one-dimensional space.

## Methods

### Definition of wave-induced energy, momentum and mass

**Figure 2:**
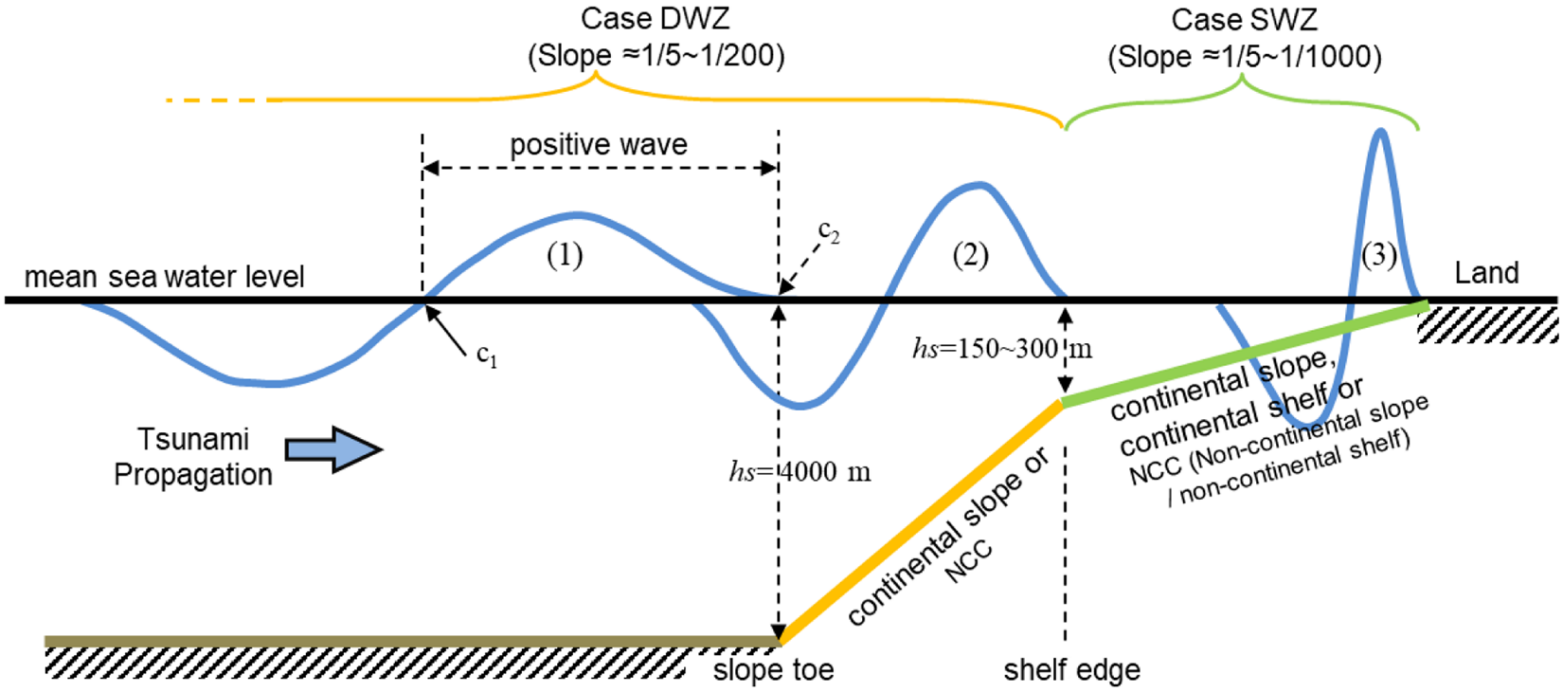
Schematic of seafloor geometry and tsunami wave. Blue lines are water surface profiles at different stages arriving at the (1) slope toe, (2) shelf edge and (3) shoreline. Refer to Table 1 for the other legend.

Although the entire lifetime of a tsunami (from its generation to the final energy dissipation near the shoreline) is of general interest, concern is often focused on how a large tsunami is triggered by an earthquake and how much of its energy (or the mass of water elevated by the earthquake) from the source region will be eventually transported to the shoreline. Accordingly, this study investigates the physical quantities of a tsunami by tracking the leading front of a tsunami travelling from the deep ocean to a shoreline as if applying a Lagrangian frame rather than observing them as they pass through a stationary point. Figure 2 schematizes a leading-elevation N-wave tsunami with idealized geometry, where the positive wave of the tsunami is most likely responsible for coastal hazards, as exemplified in Fig. 1. Physical quantities such as the energy, mass (or volume) and momentum of the positive wave of the leading-elevation N-wave are introduced to quantitatively describe the leading wave evolution.

The depth-integrated horizontal direction momentum is given by

1 $${m}_{o}=\,{\int }_{-h}^{\zeta }\rho udz$$

where ζ is the water surface elevation, *h* is the distance from the mean sea water level to bottom, *ρ* is the water density, *u* is the horizontal flow velocity and *z* is the vertical axis. The depth-integrated, wave-induced potential energy relative to the mean sea water level (*z* = 0) is given by

2 $${e}_{\varphi }={\int }_{0}^{\zeta }\rho gzdz$$

where *g* is the gravitational acceleration. The depth-integrated kinetic energy is given by

3 $${e}_{k}=\,{\int }_{-h}^{\zeta }\frac{1}{2}\rho {U}^{2}dz$$

where ${U}^{2}={u}^{2}+{w}^{2}$, and $w(=\,-\,z\partial u/\partial x-\partial hu/\partial x)$^11^ is the vertical flow velocity.

Owing to the small vertical to horizontal length scale aspect ratio (*μ* << 1), the same dimensionless variables and scale parameters as those used in the Boussinesq-type model^11^ can be employed, which results in the *O*(*μ*^2^) closure equation, as follows

4 $$\begin{array}{rcl}{m}_{o} & = & \rho (\zeta +h){u}_{\alpha }\\  &  & +\rho \{\frac{1}{2}{z}_{\alpha }^{2}(\zeta +h)-\frac{1}{2}({\zeta }^{3}+{h}^{3})\}h{u}_{\alpha xx}\\  &  & +\rho \{\frac{1}{2}{z}_{\alpha }(\zeta +h)-\frac{1}{6}({\zeta }^{2}-{h}^{2})\}{hu}_{\alpha xx}\end{array}$$

5 $${e}_{\varphi }=\frac{1}{2}\rho g{\zeta }^{2}$$

6 $$\begin{array}{rcl}{e}_{k} & = & \frac{1}{2}\rho (\zeta +h){u}_{\alpha }^{2}\\  &  & +\rho {u}_{\alpha }\{\frac{1}{2}{z}_{\alpha }^{2}(\zeta +h)-\frac{1}{6}({\zeta }^{3}+{h}^{3})\}{u}_{\alpha xx}\\  &  & +\rho {u}_{\alpha }\{{z}_{\alpha }(\zeta +h)-\frac{1}{2}({\zeta }^{2}-{h}^{2})\}h{u}_{\alpha xx}\\  &  & +\rho \{\frac{1}{6}({\zeta }^{3}+{h}^{3}){u}_{\alpha x}^{2}+\frac{1}{2}({\zeta }^{2}-{h}^{2}){u}_{\alpha x}h{u}_{\alpha x}+\frac{1}{2}(\zeta +h)h{u}_{\alpha x}^{2}\}\end{array}$$

where *u*_*α*_ is the horizontal flow velocity at *z* = *z*_*α*_, *z*_*α*_ is an arbitrary level, and the subscript *x* stands for the differential operator.

To track the energy, momentum and volume of the moving positive waves, we integrate equations (4)~(6) over [*c*_1_, *c*_2_] (in Fig. 2), as follows

7 $${M}_{o}={\int }_{{c}_{1}}^{{c}_{2}}{m}_{o}dx$$

8 $${E}_{\varphi }={\int }_{{c}_{1}}^{{c}_{2}}{e}_{\varphi }dx$$

9 $${E}_{k}={\int }_{{c}_{1}}^{{c}_{2}}{e}_{k}dx$$

10 $${V}_{w}={\int }_{{c}_{1}}^{{c}_{2}}dx$$

where *V*_*w*_ is the volume of the positive wave above the mean sea water level, equivalent to the mass of the positive wave by multiplying it by *ρ*. *c*_1_ and *c*_2_ define the tail and front faces of the positive wave, respectively, as depicted in Fig. 2.

### Critical travelling time

To assess the effect of geometry on the transmissive or reflective properties of the energy, mass (or volume) and momentum of travelling tsunami waves in later sections of this study, a critical travelling time (*T*_*c*_) is introduced. The infinitesimal time (*dt*) spent for a wave propagating over a distance of *dx* is given by

11 $$dt=\frac{dx}{\sqrt{gh(x)}}$$

where *x* is the cross-shore distance from the toe to a local point on a sloped plain. Then, *T*_*c*_ for a tsunami travelling from the toe (*x* = 0) to the shoreline (*x* = *h*_*x*_/*S*) is derived as follows

12 $${T}_{c}={\int }_{0}^{{h}_{s}/S}\frac{1}{\sqrt{g({h}_{s}-xS)}}dx=\frac{2}{S}\sqrt{\frac{{h}_{s}}{g}}$$

where *h*_*s*_ is the water depth at the toe, *S* is the slope of the plain, and *h*(*x*)(=*h*_*s*_ − *xS*) is the water depth at the local point. Therefore, the average celerity of the wave travelling from the toe to the shoreline is $\sqrt{g{h}_{s}}/2$.

### Flow model

Normally, tsunami events at a geophysical scale have very small vertical to horizontal length scale aspect ratios and wave height to water depth ratios; thus, it is not easy to measure wave propagation with these ratios in the laboratory. In addition, considering that a tsunami wave is affected by complex coastal processes over uneven topography in nature, the application of numerical methods based on fundamental flow equations can be an appropriate approach. Boussinesq-type models can simulate tsunami motion and surf zone processes from deep to intermediate and shallow waters^6,11^. Here, a Boussinesq-type model for weakly dispersive, rotational and turbulent flow including the wave breaking dissipation effect and moving boundary scheme^11,12^ is employed, as follows

13 $$\frac{\partial \zeta }{\partial t}+\frac{\partial H{u}_{i}}{\partial {x}_{i}}+M+{M}^{\nu }=0$$

14 $$\begin{array}{c}\frac{\partial H{u}_{i}}{\partial t}+\frac{\partial H{u}_{i}{u}_{j}}{\partial {x}_{j}}+gH\frac{\partial \zeta }{\partial {x}_{i}}+H({D}_{i}+{D}_{i}^{\nu }+{\bar{\xi }}_{i}+\,{\bar{\xi }}_{i}^{\nu })+{u}_{i}(M+{M}^{\nu })\\ -H\frac{\partial }{\partial {x}_{j}}(2{\nu }_{t}^{h}{S}_{ij})+2H\frac{\partial }{\partial {x}_{i}}({\nu }_{t}^{v}\frac{\partial {u}_{j}}{\partial {x}_{j}})+\frac{{\tau }^{b}}{\rho }+H{R}_{i}^{b}=0\end{array}$$

where *i*, *j* = (1, 2). *x*_*i*_ represents the spatial axes. *t* is time, ${u}_{i}=(u,v)$ is the flow velocity, and *H* = (ζ + *h*) is total water depth. ${\nu }_{t}^{v}$ and ${\nu }_{t}^{h}$ are the vertical and horizontal eddy viscosities, respectively. *M* and *M*^*v*^ are the second-order dispersion and vorticity correction terms. *D*_*i*_ and ${D}_{i}^{v}$ are the frequency dispersion correction terms due to the wave and turbulence generated at the bottom boundary layer, respectively. ${\bar{\xi }}_{i}$ and ${\bar{\xi }}_{i}^{\nu }$ are the vorticity correction terms due to the wave and turbulence, respectively. More detailed expressions for these higher-order terms are described in Kim *et al*.^11^. The bottom friction term, ${\tau }_{i}^{b}/\rho$, is modelled using the Manning formula, where the Manning friction coefficient is given by *n* = 0.013. ${R}_{i}^{b}$ is the wave-breaking dissipation term^13^. A detailed description of the numerical schemes is provided in the Supporting Information (SI).

### Wave and geometric configurations

Typical ocean bathymetry can be simplified as an abyssal plain, a continental rise, a continental slope^14^ and a continental shelf^15^. Although these four bathymetric features are connected, as shown in Fig. 2, this continuous bathymetry setup may result in numerous simulation cases and complicated analyses when considering different bottom slopes of the continental slope and continental shelf, various depths of the continental shelf edge, or water depth of the tsunami generation location. In this study, we classified the ocean geometry into two undersea zones, the DWZ (Deep Water Zone) and SWZ (Shallow Water Zone), as shown in Fig. 2, since they seem to have distinctive impacts on the tsunami evolution and transmission.

Both DWZs and SWZs comprise a flat seabed and a plane slope. A DWZ denotes a relatively deep and steeply sloped bathymetry including a continental slope. For the DWZ cases, the incident tsunami is generated on the flat seabed where *h*_*s*_ = 4000 *m*, and the physical quantities of interest are calculated from when the positive wave front (*c*_2_) is on the slope toe to when the front reaches the shelf edge, where *h*_*s*_ = 200 m. A SWZ denotes a single slope at relatively shallow water area including a continental shelf, NCC or continental slope. For the SWZ cases, the incident wave is generated on a flat seabed where *h*_*s*_ = 150~300 *m*, and the physical quantities are calculated from when the positive wave front is at the slope/shelf edge to when the front reaches the shoreline. Considering the average slopes of natural continental slopes and continental shelfs, which are approximately 1/15 and 1/570, respectively^14,15^, the bottom slopes of DWZs and SWZs are given by *S* = 1/5~1/200 and *S* = 1/5~1/1000, respectively. Table 1 summarizes the wave and geometric conditions tested in this study. It should be noted that for the analysis in later sections of this study, ‘continental slope’ denotes the geometry with *S* = 1/5~1/30 for both the DWZ and SWZ, and ‘continental shelf’ denotes the geometry with *S* = 1/300~1/1000 for the SWZ, approximately from half to double the range of the natural average.

**Table 1:** Wave and geometric configurations.

Case	DWZ (Deep Water Zone)		SWZ (Shallow Water Zone)		
Geographic feature	Continental Slope	NCC^*^	Continental Slope	NCC	Continental Shelf
Slope (*S*)	$\frac{1}{5} \sim \frac{1}{30}$	$\frac{1}{40} \sim \frac{1}{200}$	$\frac{1}{5} \sim \frac{1}{30}$	$\frac{1}{40} \sim \frac{1}{200}$	$\frac{1}{300} \sim \frac{1}{1000}$
Depth of toe (*h*_*s*_)	4000 m		150 m, 200 m, 300 m		
Wave period (*T*_*w*_)	780 s, 1560 s				
Wave amplitude (*a*_*o*_)	1 m, 2 m		2 m, 4 m		

*NCC: Non-continental slope/non-continental shelf.

Conventionally, tsunamis have been modelled as solitary wave, N-wave or a combination of solitary waves. Meanwhile, Madsen *et al*.^6^ pointed out that solitary wave was barely justified for geophysical scale tsunami modelling just as meaningful evidence supporting the difference between N-wave and solitary wave has been reported^16–19^. Accordingly, we generate a single-period sinusoidal wave on the flat seabed, which can be found through recent tsunami-related works^20–22^. The incident wave is designed to be $\zeta (t)={a}_{o}\,\sin (2\pi t/{T}_{w})$ with $0\le t\le {T}_{w}$. By considering the geophysical scale in the field^6^, the wave periods are given by *T*_*w*_ = 780 *s* and 1560 *s*, while the wave amplitude is given by *a*_*o*_ = 1 *m* and 2 *m* for the DWZ and *a*_*o*_ = 2 *m* and 4 *m* for the SWZ. These periods and heights are determined not only to consider the feasibility of actual events but also to ensure the generality of the perspective results. It should also be noted that the value of *a*_*o*_ of the SWZ being double that of the DWZ is selected based on preliminary numerical tests, which roughly evaluated that the wave amplitude at the slope/shelf edge after the shoaling process from the continental slope toe to the continental shelf is approximately 2 times the value of *a*_*o*_ of the DWZ.

## Results

### Mass, momentum and energy paradigm of travelling tsunami

In this section, transmissive ratios ${{\mathscr{T}}}_{Mo}$, ${{\mathscr{T}}}_{E\varphi }$, ${{\mathscr{T}}}_{Ek}$, and ${{\mathscr{T}}}_{Vw}$ are defined from the quantities of *M*_*o*_, *E*_*ϕ*_, *E*_*k*_, and *V*_*w*_ of a tsunami at a normalized depth, ${h}_{n}(=\,h(x)/{h}_{s})$, relative to the corresponding quantities evaluated at stage (1) for DWZ, and at stage (2) for SWZ in Fig. 2, respectively.

**Figure 3:**
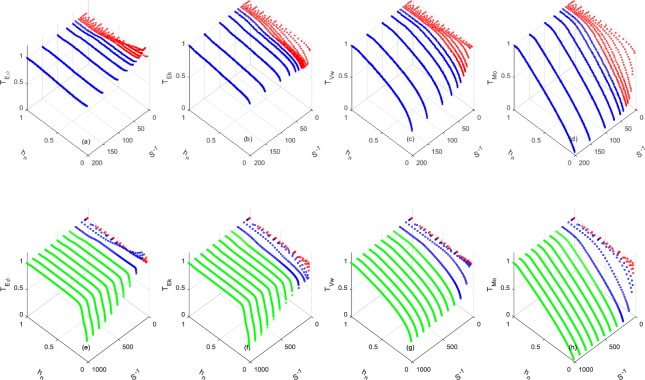
(**a**~**d**) Transmissive ratio for the DWZ with *a*_*o*_ = 1 *m*, *T*_*w*_ = 780 s, and *h*_*s*_ = 4000 *m*. (**e**~**h**) Transmissive ratio for the SWZ with *a*_*o*_ = 2 *m*, *T*_*w*_ = 780 s, and *h*_*s*_ = 200 *m*. Green: Continental shelf. Blue: NCC. Red: Continental slope.

Figure 3(a~h) show the variation in the transmissive ratios of tsunamis travelling on DWZ and SWZ, respectively. (Readers can refer to Figs S1 and S2, which are equivalent to the side views of Fig. 3). For both the DWZ and SWZ cases, ${{\mathscr{T}}}_{Vw}$ and ${{\mathscr{T}}}_{Mo}$ decrease as waves *h*_*n*_ decreases. Meanwhile, ${{\mathscr{T}}}_{Ek}$ on the continental slope and continental shelf show somewhat different patterns; both are conserved well, up to a certain depth of *h*_*n*_. However, thereafter, ${{\mathscr{T}}}_{Ek}$ on the continental slope and NCC decreases slightly for either the DWZ or SWZ, but ${{\mathscr{T}}}_{Ek}$ on the continental shelf decreases drastically. ${{\mathscr{T}}}_{E\varphi }$ on the continental slope and shelf exhibit opposite behaviours. ${{\mathscr{T}}}_{E\varphi }$ is conserved well initially, but the conservation breaks down beyond a certain depth, showing that ${{\mathscr{T}}}_{E\varphi }$ on the continental slope increases slightly, while ${{\mathscr{T}}}_{E\varphi }$ on the continental shelf decreases significantly.

**Figure 4:**
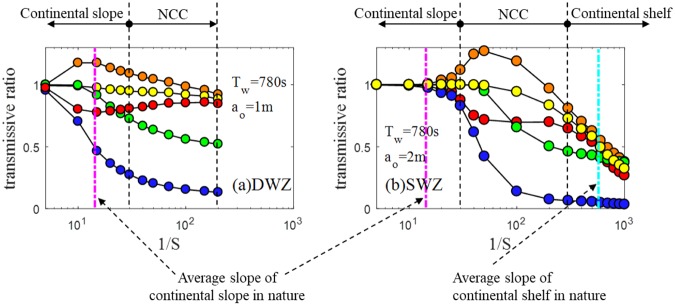
(**a**) Transmissive ratios for the DWZ at the shelf edge. (**b**) Transmissive ratios for the SWZ at the shoreline (*h*_*s*_ = 4000 m). Orange: ${{\mathscr{T}}}_{E\varphi }$, red: ${{\mathscr{T}}}_{Ek}$, green: ${{\mathscr{T}}}_{Vw}$, blue: ${{\mathscr{T}}}_{Mo}$, yellow: $1/2({{\mathscr{T}}}_{E\varphi }+{{\mathscr{T}}}_{Ek})$. Vertical magenta and cyan lines represent the average *S* values of continental slopes and continental shelfs, respectively, in nature. Vertical black dashed lines show the range of double the average *S*.

Figure 4(a,b) show the transmissive ratios at the shelf edge and shoreline, respectively, and some implications may be drawn out. First, all the results decrease upon reaching the shoreline across the continental shelf, as *S* becomes more moderate, as shown in Fig. 4(b). This is consistent with field observations and is difficult to support using the results of studies conducted on non-geophysical scales. Second, on continental slopes in both the DWZ and SWZ cases, ${{\mathscr{T}}}_{Mo}$ and ${{\mathscr{T}}}_{Vw}$ decrease continuously as *S* decreases. However, ${{\mathscr{T}}}_{E\varphi }$ increases and even develops over unity, which seems to occur due to the fairly short distance for wave transformation on the sloping bathymetry as well as due to the abrupt change in the bottom shape (Fig. S3). ${{\mathscr{T}}}_{Ek}$ shows an opposite tendency to that of ${{\mathscr{T}}}_{E\varphi }$, which results from the energy transformation discussed in a later section of this study. Cases other than those in Fig. 4 are presented in Fig. S4 and show patterns quite consistent with the findings observed in Fig. 4.

Although the tested cases cover a wide range of *S* for research purposes, it is meaningful to analyse the result when *S* corresponds to a realistic value. Within the range of ‘continental slope’, ${{\mathscr{T}}}_{E\varphi }\approx 1.0\, \sim \,1.2$, ${{\mathscr{T}}}_{Vw}\approx 0.7\, \sim \,1.0$, ${{\mathscr{T}}}_{Ek}\approx 0.8\, \sim \,1.0$, and ${{\mathscr{T}}}_{Mo}\approx 1.0\, \sim \,0.3$. This demonstrates that most of the volume of water elevated at the source region is possibly delivered to the continental shelf edge, so the potential and kinetic energy transfer very efficiently. In addition, it is obvious that the potential energy is the strongest factor driving tsunami hazards to where a steep plane extends from the deep seabed to the near-coast. Within the range of ‘continental shelf’, the results exhibit much lower ${{\mathscr{T}}}_{E\varphi }$, ${{\mathscr{T}}}_{Ek}$, ${{\mathscr{T}}}_{Vw}$, and ${{\mathscr{T}}}_{Mo}$ compared to those at the ‘continental slope’, which indicates that fairly long and mild-sloped continental shelfs are capable of effectively protecting coastal areas from tsunami events.

Additionally, transmissive properties are relatively insensitive to different configurations of wave in that nearly identical shoaling processes may be expected even with different wave shapes at the slope toe. For example, doubling the wave height offshore is found to have limited effects on the transmissive ratio, according to Fig. S1(a,e). Therefore, the physical attenuation or amplification of tsunami properties primarily depends on bathymetry rather than on wave conditions under the tested geophysical scale.

**Figure 5:**
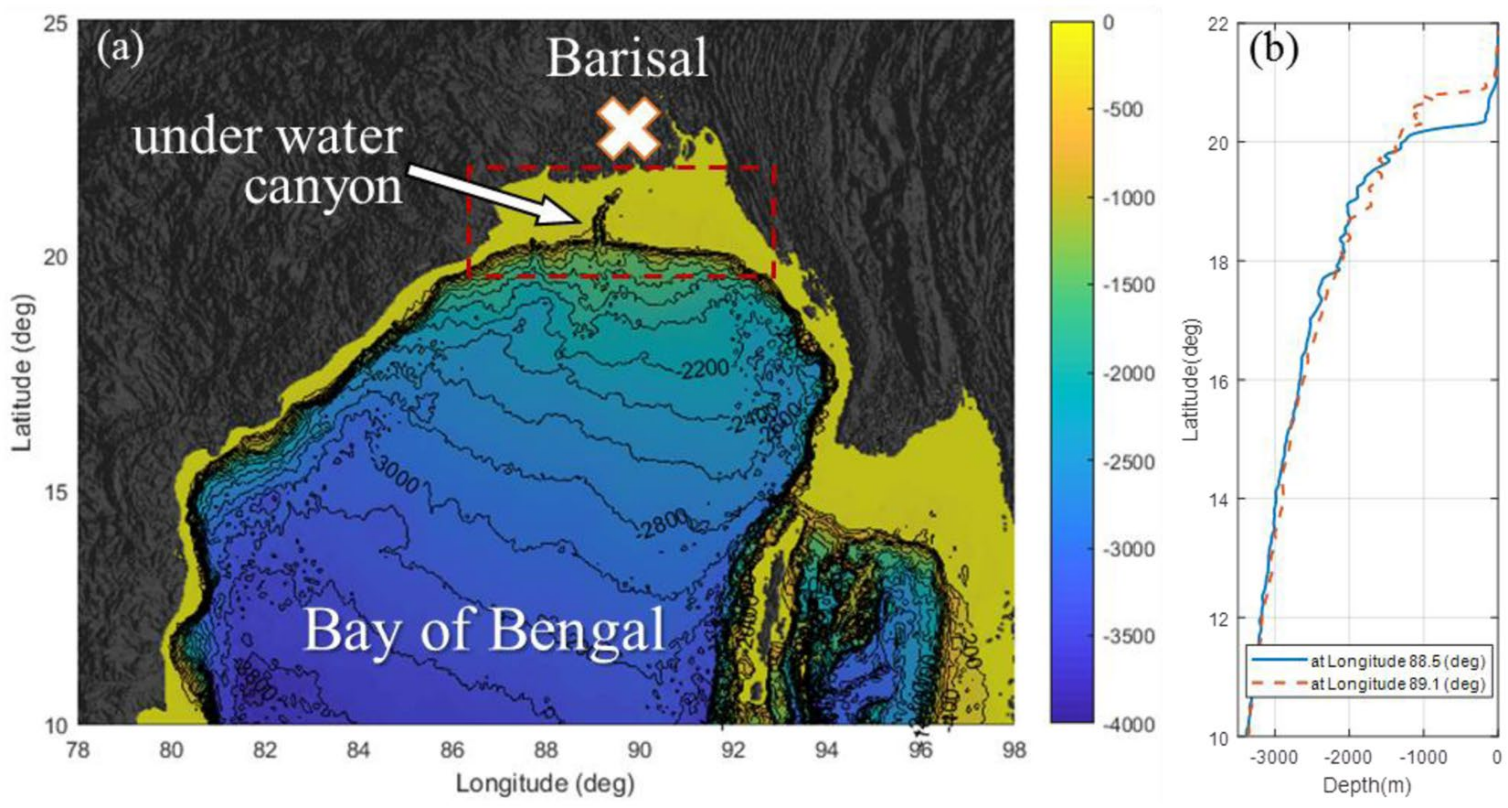
(**a**) Coastal relief around the Bay of Bengal. Red dashed box denotes the continental shelf area. Grey represents land. Colormap (and contour) represents the water depth in metres. (**b**) Water depth profile from deep sea to coast.

Figure 5 shows the bathymetry around the Bay of Bengal, where a relatively uniform continental slope and shelf are formed, except in the undersea canyon. The tsunami that occurred on 26 December 2004 in the Indian Ocean severely damaged most of the coasts of the Bay of Bengal except the coast of Bangladesh, where the continental shelf with a very mild slope of *S* ~ 0.001 extends far into the ocean. Exceptionally, two casualties were reported only around Barisal, which is connected through the undersea canyon to the toe of the continental slope^23^. Ioualalen *et al*.^24^ simulated the tsunami event using a Boussinesq-type model for the area and found that the geometry of the continental shelf of Bangladesh damped the tsunami wave; however, at the underwater canyon, the slope of the bathymetry is relatively steep compared with the other coastal areas of Bangladesh, and the damping was not the same in this area. Determining a reasonable explanation for this event is possible using the proposed paradigm, although the proposed results in Figs 3 and 4 cannot be directly applied to the observed cases because there is no continuously measured data from the source to the coast in the cross-shore direction.

### Tsunami damping mechanism

**Figure 6:**
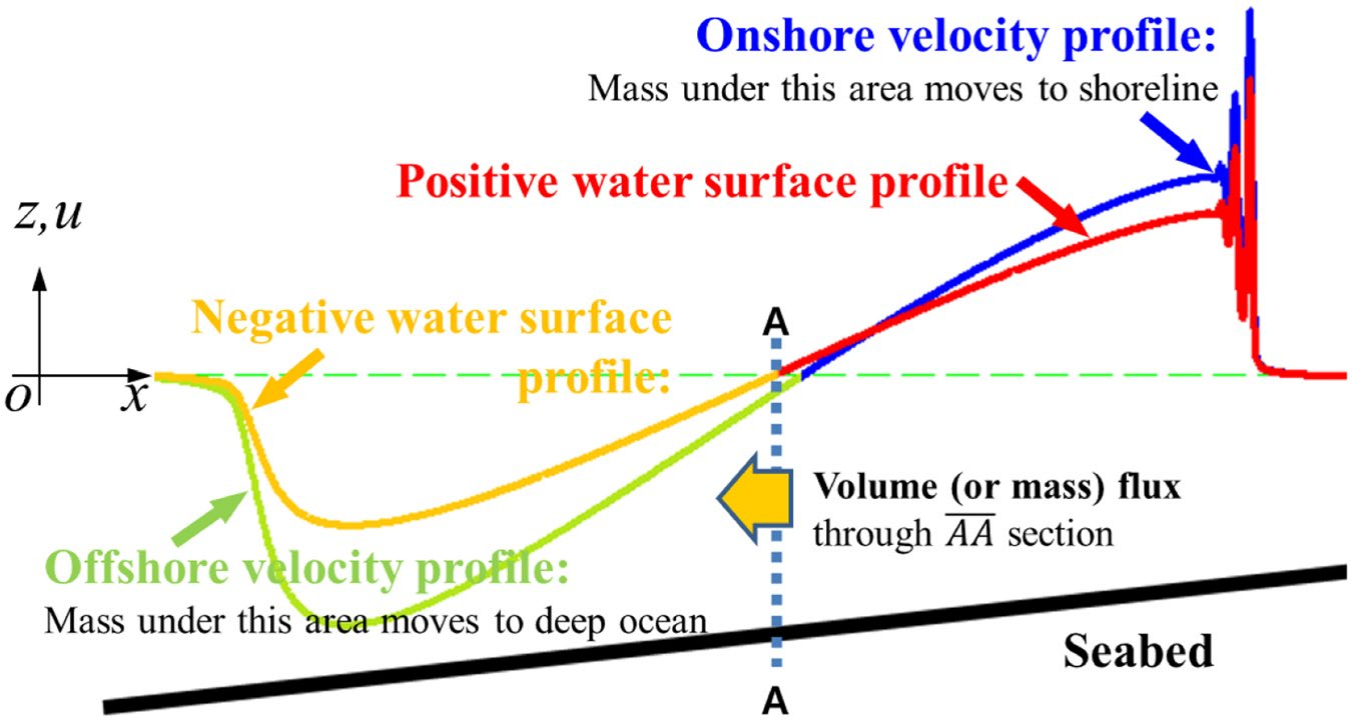
Schematic of the volume reduction process. Green dashed line: still water level. Section $\overline{AA}$: interface between positive and negative water surface profiles.

**Figure 7:**
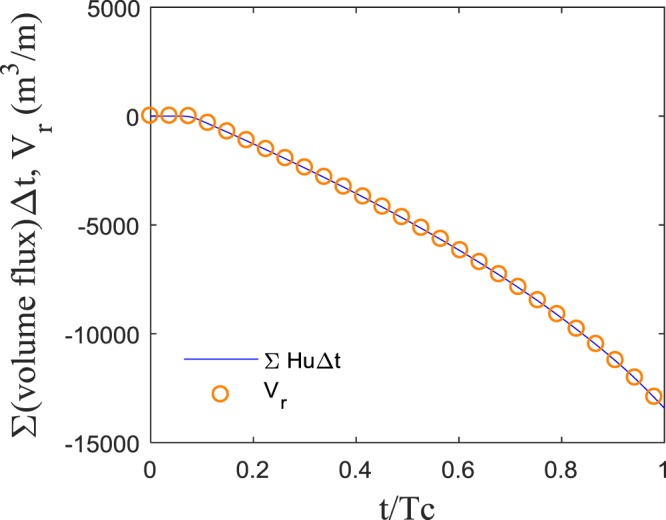
Comparison of accumulated volume flux through $\overline{AA}$ and *V*_*r*_(=−Δ*V*_*w*_) per unit width for *S* = 1/600, *a*_*o*_ = 2 *m*, and *h*_*s*_ = 200 *m*.

Considering that the positive wave is surrounded by air, the shoreline and sea bottom, the only outlet of the travelling positive wave is section $\overline{AA}$ (where $\zeta =0$), as shown in Fig. 6. Due to the asymmetry of the wave profile, there are locations where $\zeta =0$ and *u* = 0 do not coincide. Throughout the studied cases, we confirmed that all the computed *u* at $\overline{AA}$ were headed towards the offshore along the slopes. Therefore, there can be a cross-shore volume flux through $\overline{AA}$, and the decrease in *V*_*w*_ should be the same as the accumulated volume flux through $\overline{AA}\,(=\,{\iint }_{H}udzdt)$, as shown in Fig. 7. Consequently, a damping mechanism for the tsunami volume is explained through the volume flux. As shown in Fig. 4(b), $(1-{{\mathscr{T}}}_{Vw})$ on the continental shelf is significantly larger than on the continental slope. That is, much larger volume is reflected by the continental shelf than by the continental slope.

**Figure 8:**
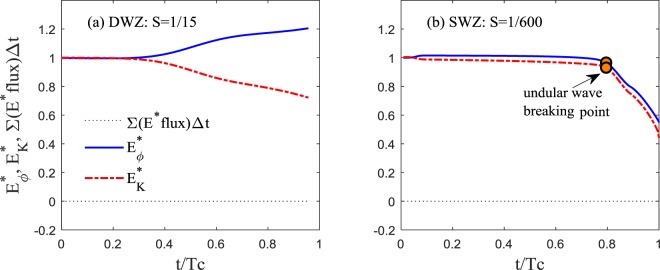
Energy evolution (per unit width). (**a**) *T*_*w*_ = 780 s and *a*_*o*_ = 1.0 *m*. (**b**) *T*_*w*_ = 780 s, *a*_*o*_ = 2 m, and *h*_*s*_ = 200 *m*. Superscript * represents normalization by *E* of the initial positive wave.

The depth-integrated wave-induced potential energy flux based on the Boussinesq approximation, *flux*_*Eϕ*_, is given by

15 $$\begin{array}{rcl}Flu{x}_{E\varphi } & = & \rho g(\frac{1}{2}{\zeta }^{2}+\zeta h){u}_{\alpha }\\  &  & +\rho gh(\frac{1}{2}{z}_{\alpha }^{2}\zeta -\frac{1}{6}{\zeta }^{3}){u}_{\alpha xx}+\rho gh({z}_{\alpha }\zeta -\frac{1}{2}{\zeta }^{2})h{u}_{\alpha xx}\\  &  & +\rho g(\frac{1}{4}{z}_{\alpha }^{2}{\zeta }^{2}-\frac{1}{8}{\zeta }^{4}){u}_{\alpha xx}+\rho g({z}_{\alpha }{\zeta }^{2}-\frac{1}{3}{\zeta }^{3})h{u}_{\alpha xx}\end{array}$$

and becomes zero at $\overline{AA}$ (where $\zeta =0$). This is very interesting because a propagating long wave maintains its potential energy even over an uneven sea bottom if neglecting the energy loss from wave breaking, bed friction and turbulence up to *O*(*μ*^2^). This finding can also be supported by the simulation results that show extremely small amounts of accumulated energy flux through $\overline{AA}$, as in Fig. 8. Therefore, it is difficult to argue that energy flux can entirely explain the cause of damping or amplification.

The simulations on continental slopes show that wave breaking does not occur, and part of *E*_*k*_ is converted to *E*_*ϕ*_ as the wave approaches the shelf edge, as shown in Figs 3 and 8(a). For the wave on a continental shelf, we observed the wave disintegrating into shorter, breakable secondary waves when *S* < 1/200. In addition, as *S* decreases, the distance between the beginning point of the undular bore and shoreline becomes longer, enough for wave breaking to play a role in damping the energy. As a result, *E*_*ϕ*_ and *E*_*k*_ rapidly drop after breaking occurs on the undular bores (Figs 3 and 8(b)). When *S* > 1/200, strong breaking is not observed, and the transmissive ratio remains high. Madsen *et al*.^6^ conducted a similar numerical test of geophysical scalewith *S* = 1/200 and reported that no undular bore was observed.

**Figure 9:**
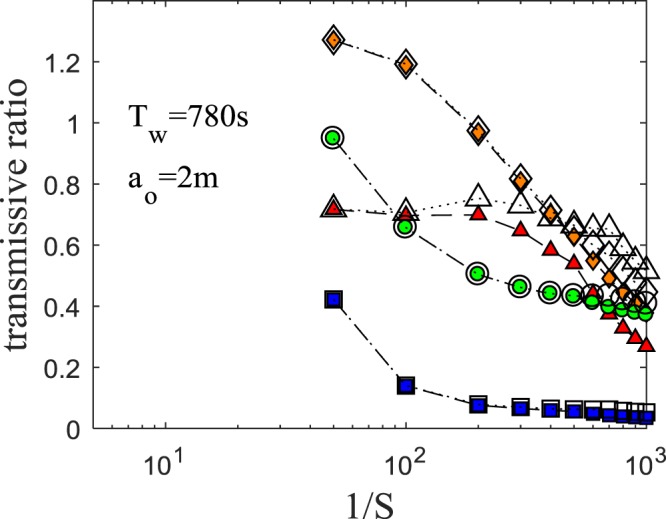
Comparison of transmissive ratio. Coloured symbol: with friction terms. White symbol: without friction terms. Diamond: ${{\mathscr{T}}}_{E\varphi }$. Triangle: ${{\mathscr{T}}}_{Ek}$. Circle: ${{\mathscr{T}}}_{Vw}$. Square: ${{\mathscr{T}}}_{Mo}$.

To examine bottom friction effects more explicitly, we compared simulation results with and without friction terms in Fig. 9 (and Fig. S5 in SI). Without friction terms, there is still a significant reduction of energy, mass and momentum. The transmissive ratios without friction terms show slightly higher values compared to those as a result of including the friction effects. This can be verified analytically. Liu *et al*.^25^ proposed an equation for frictional damping:

16 $${(\frac{a}{{a}_{o}})}^{-1/4}=1+0.08356(1+\frac{h}{W}){(\frac{\nu }{h\sqrt{gh}})}^{1/2}{(\frac{{a}_{o}}{h})}^{1/4}(\frac{x}{h})$$

where *a* is the wave amplitude, *a*_*o*_ is the incident wave amplitude, *W* is the width of the channel and *ν* is the viscosity. Substituting the average length of the continental shelf (65 *km*)^15^, depth (20~200 *m*) and a typical *a*_*o*_ (1 *m*) into equation (14) results in less than 3% attenuation. Although the contribution of bottom friction is not significant, the effects will grow with respect to *S*^−1^ (equivalently, the shelf length) and bottom roughness. Note that the effects of friction on runup height should be more considerable, even though we do not present the results in this study.

## Conclusions

In this study, we presented a new paradigm describing tsunami-induced potential energy, kinetic energy, mass, and momentum. Such physical quantities are presented on a Lagrangian-like frame by following the moving volume of a positive wave, not by observing them at fixed locations. To provide geophysical explanations of the relationship between typical sea bathymetry and tsunami evolution, we created two typical bathymetry groups representing typical examples of continental slopes and continental shelfs with average features from natural geometries. A geophysical scale wave condition was adopted, and a fully nonlinear, weakly dispersive, rotational, and turbulent flow model was used for the numerical simulation. The major findings from the analysis are summarized as follows.

1. The results of the suggested Lagrangian-like volume tracking paradigm is consistent with observations from nature:
  As mentioned above, it is found in many studies that steeply sloped bathymetry can protect coastal areas from tsunamis, contrary to the field observations. Nevertheless, the methods and analyses used in previous research are very reasonable and sound; thus, we presume that the contradictory results result from different viewpoints on how we observe or what we measure. In terms of the viewpoint, the majority of conventional studies on tsunami evolution on sloped bathymetry have focused on the physics at fixed points. In contrast, in this study, we chose and quantified the representative physical factors of a travelling tsunami not based on local points but based on the moving volume. As a result, the proposed paradigm is consistent with the field observations. For example, applying this paradigm to the eastern coasts of Japan and China results in higher and lower tsunami hazards, respectively.
2. Energy transformation plays an important role on steep slopes:
  ${{\mathscr{T}}}_{E\varphi }$ and ${{\mathscr{T}}}_{Ek}$ passing the continental slope and continental shelf show different patterns. On the shelf bathymetry, *E*_*ϕ*_ and *E*_*k*_ are conserved well before breaking occurs, indicating that the energies do not transform well. After breaking occurs, *E*_*ϕ*_ and *E*_*k*_ drop simultaneously, which is certainly not due to energy transformation but rather due to the dissipation process. On continental slopes, part of *E*_*k*_ transforms to *E*_*ϕ*_; thus, ${{\mathscr{T}}}_{Ek}$ decreases more than ${{\mathscr{T}}}_{E\varphi }$, and the dissipation is very small.
3. Undular bore breaking can be a turning point of the energy paradigm on a long continental shelf:
  Before a wave (specifically, undular bore) breaks, the wave energy on an uneven bottom is maintained from the viewpoint of the newly proposed paradigm when ignoring energy dissipation by bottom friction and turbulence. While a tsunami is approaching the coastline, the undular bore on the main wave develops high peaks of *ζ* and *u*. These relatively short secondary waves collapse down through ‘breaking’, and then *e*_*ϕ*_ (proportional to *ζ*^2^) and *e*_*k*_ (proportional to *u*^2^) decrease more dramatically as *S* decreases. Applying this interpretation to actual events, such as that illustrated in Fig. 1, will result in a substantial amount of tsunami energy delivered to the shoreline because the wave does not break. On the other hand, applying this interpretation to vast continental shelfs will result in a reduced transmissive energy due to wave breaking.
4. The longer (and milder) the sea bottom, the more water is released offshore:

On slopes, the locations where the flow velocity and water surface elevation equal zero are separated. As a result, $\overline{AA}$ acts like the outlet of the elevated water volume, and its volume (or mass) reduces as much as the time-integrated flux. Given this notion, as *T*_*c*_ lengthens (equivalently, as *S* decreases), *V*_*r*_ increases; thus, ${{\mathscr{T}}}_{Vw}$ decreases. It is interesting to see that the $(1-{{\mathscr{T}}}_{Vw})$ on the continental shelf is remarkably larger than that on the continental slope for typical natural geometries. The smaller the transmitted volume, the less inundation or overtopping occurs.

In addition, the case without bottom friction terms also shows a significant reduction of the transmissive ratios, implying that a certain portion of tsunami damping originates from the topography itself rather than the bottom friction. However, when $S\lesssim 1/800$, the dissipation terms begin to contribute to some degree. Thus, bottom friction partially contributes to reduce tsunami hazards along very long and flat continental shelfs.

Although the proposed results provided several outputs and conclusions, the limitation of this work is clear: the bathymetry and incident wave are highly idealized. Coral reefs, ripples and other complex bedforms can increase the fictional effects. In addition, there are many additional influences such as fault direction, refraction, and source location, which is not considered in this work.

## Electronic supplementary material

Supplementary Information
