# Supplementary material for: Lagrangian-like Volume Tracking Paradigm for Mass, Momentum and Energy of Nearshore Tsunamis and Damping Mechanism
Source: Sci Rep. 2018 Sep 21;8:14183. doi: 10.1038/s41598-018-32439-6 (PMC6155013; doi:10.1038/s41598-018-32439-6)
Supplement: Supplementary file 1 — Supplementary Information [file 41598_2018_32439_MOESM1_ESM.pdf]

Supporting Information for

Lagrangian-like Volume Tracking Paradigm for Mass, Momentum and Energy of Nearshore Tsunamis and Damping Mechanism

[Dae-Hong Kim[1] and Sangyoung Son[2]]

[1]Department of Civil Engineering, University of Seoul.

[2]School of Civil, Environmental and Architectural Engineering, Korea University.

Contents of this file

Text S1 to S2
Figures S1 to S5

Text S1. Numerical scheme

The computational time step was determined using a CFL condition: $\Delta\mathrm{t} = \mathrm{CFL} \times \min\left\{\Delta x / \left(|u| + \sqrt{gH}\right)\right\}$ with $\mathrm{CFL} = 0.5$, where $\Delta\mathrm{t}$ is the computational time step and Δx is the computational grid size. For the numerical simulation of tsunamis over DWZs and SWZs, computational grids of different sizes were used. Because the wave length of the DWZ case is very long, $\Delta x = 100$ m was used. For the numerical simulation over a SWZ, wave fission was observed, and an undular bore was created. Thus, a smaller grid size $\Delta x = 10$ m was adopted.

The timing of waves (1) and (2) in Figure 2 were determined at the instant when the water surface elevation becomes $\zeta = 0.005$ m at the slope toe and shelf edge, respectively. For wave (3) in Figure 2, a total water depth of $H = 0.005$ m was used to determine the timing. To calculate the integration of the physical variables in equations (7) ~ (10), the trapezoidal rule was used. The criterion of location utilizes the positive wave front, that is, c_2.

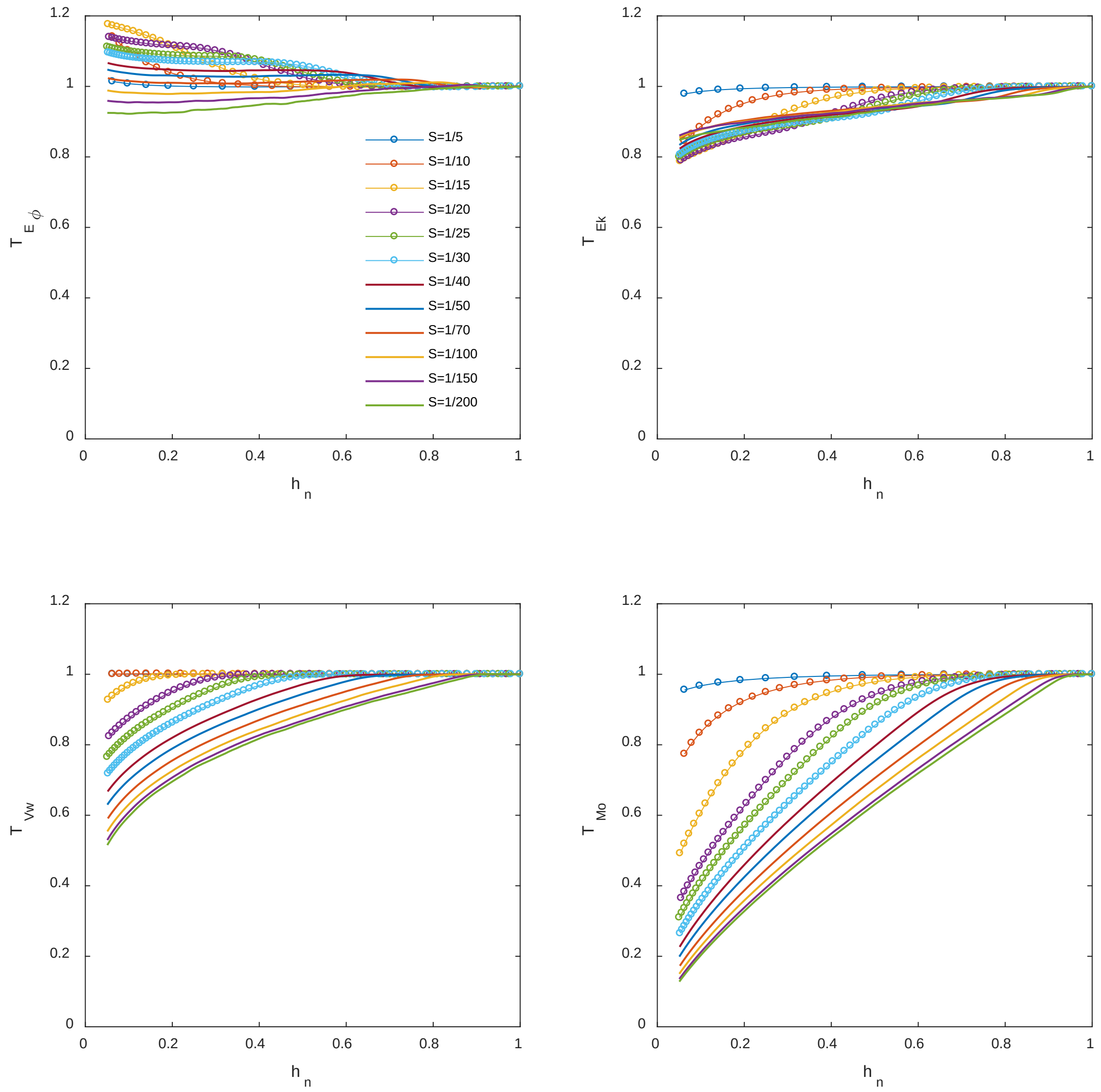

Figure S1. Transmissive ratio for DWZ: Side views of Figure 3(a~d)

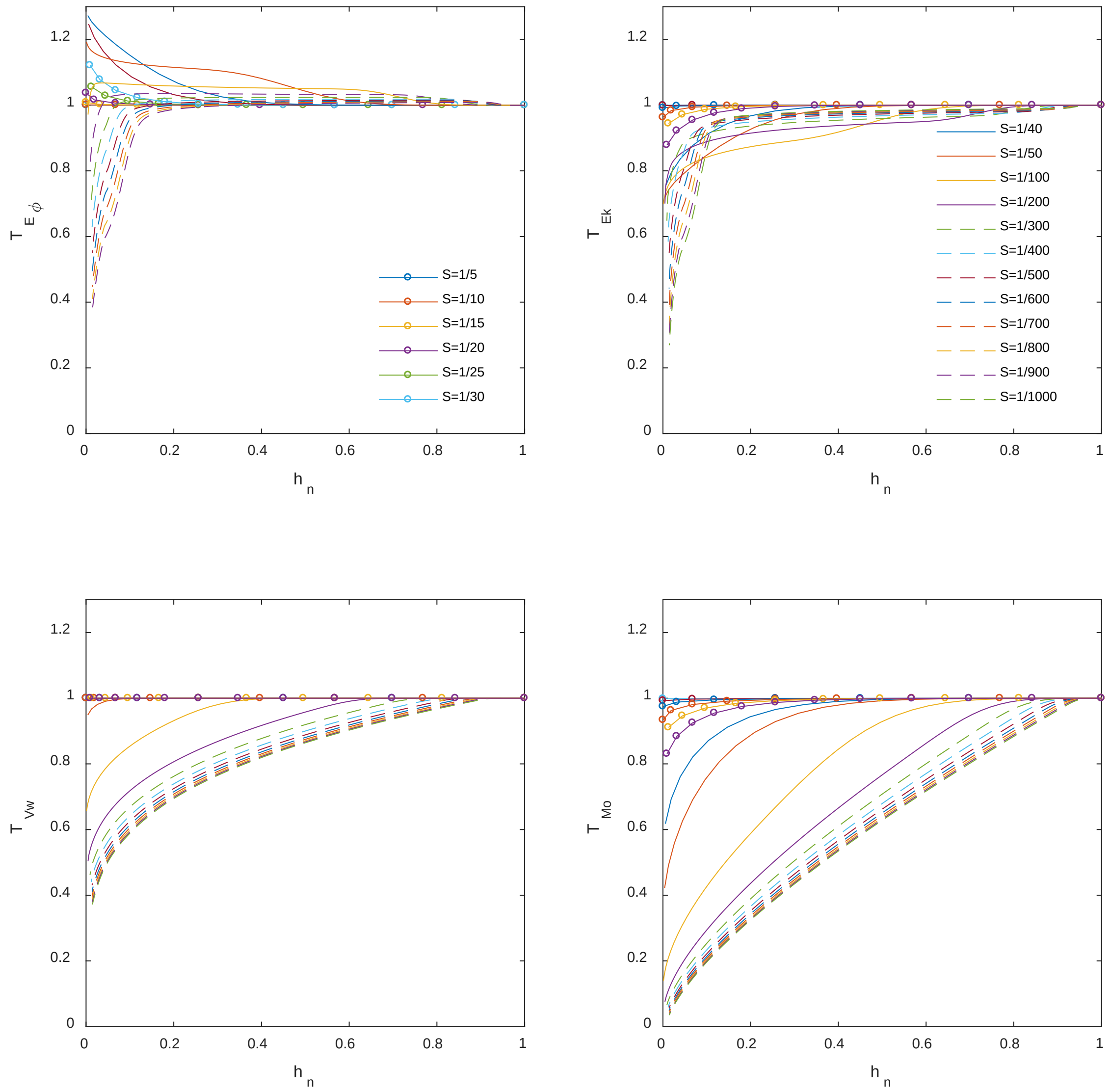

Figure S2. Transmissive ratio for SWZ: Side views of Figure 3(e~h)

Text S2. Bathymetry and wave dimension ratio

Although we suggested in the main text that h_n and S were effective factors for tsunami attenuation, they are not the only factors that govern the transmissive ratio. For example, the transmissive ratios for S = 1/200 are different for DWZ and SWZ. One of the reasons can be presumed based on the ratio between slope length (L_s) and wave length (L_w): if L_s is much shorter than the incident L_w, the tsunami may not have enough time to interact with the bathymetry before its front reaches the shoreline, as shown in Figure S2(a). If L_s is much longer than L_w, then the wave has a long chance to respond to the varying seabed geometry, as shown in Figure S2(b).

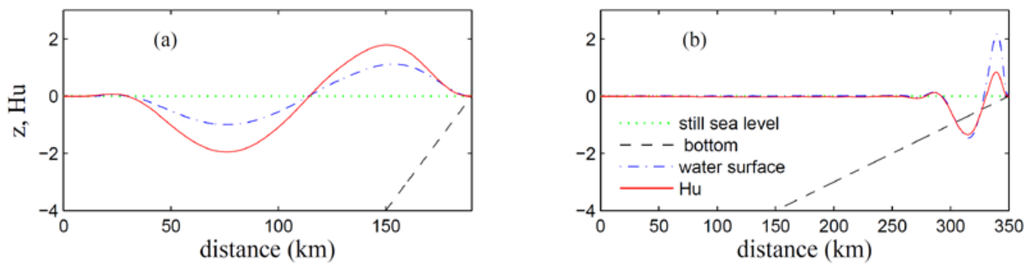

Figure S3. Snapshots of tsunami properties when the wave front reaches the shoreline. (a) S=1/10. (b) S=1/50. The units of bottom elevation, water surface elevation and discharge (Hu) are *km*, *m* and $m^2/s/100$, respectively.

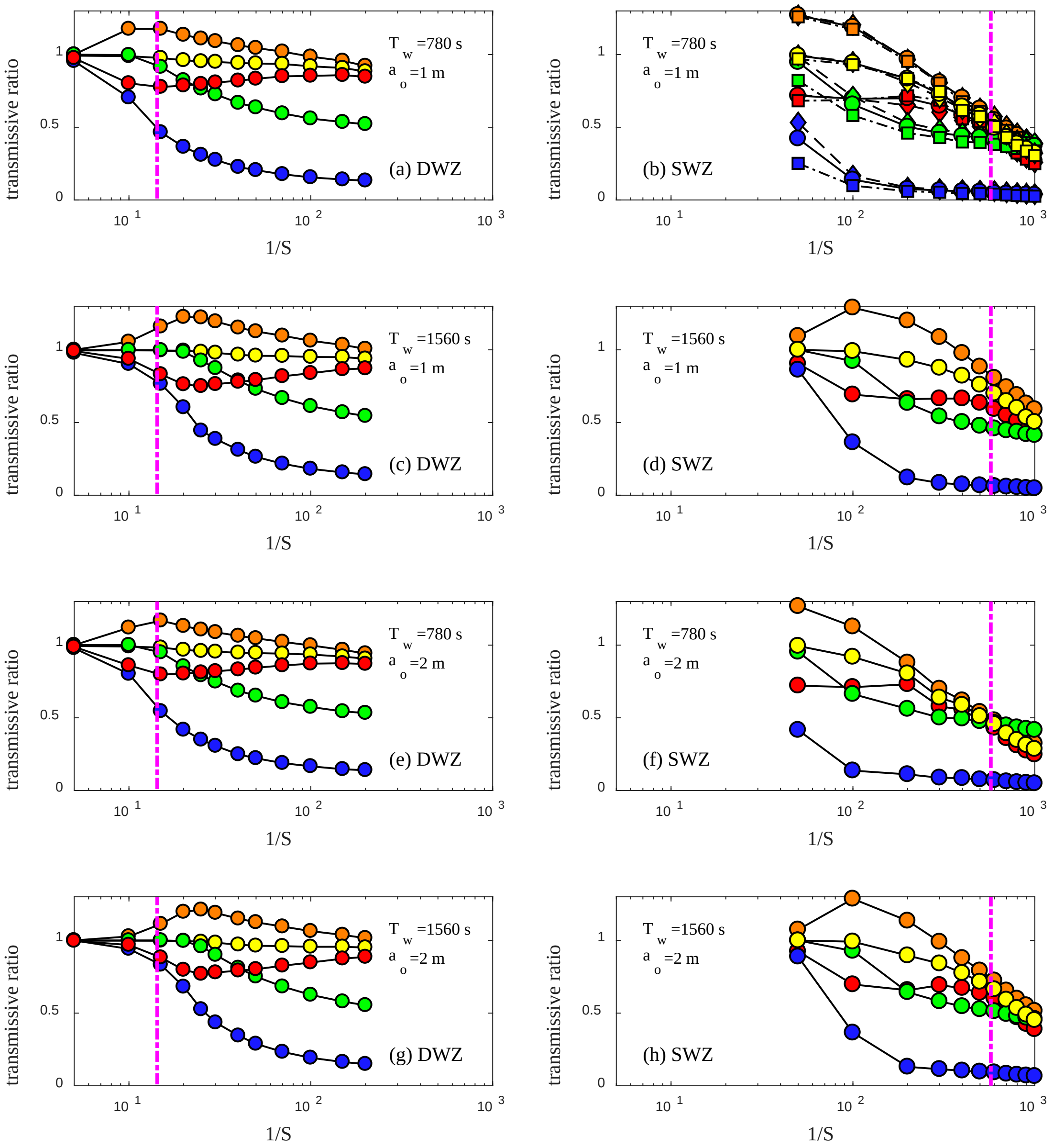

Figure S4. Transmissive ratios at stage (3) in Figure 2. Orange: $T_{E\phi}$. Red: T_{Ek}. Green: T_{Vw}. Blue: T_{Mo}. Yellow: $1/2(T_{E\phi}+T_{Ek})$. Magenta line: average S in nature. For SWZ, diamonds, circle and square stand for the cases with $h_s = 150\ m$, $200\ m$ and $300\ m$, respectively.

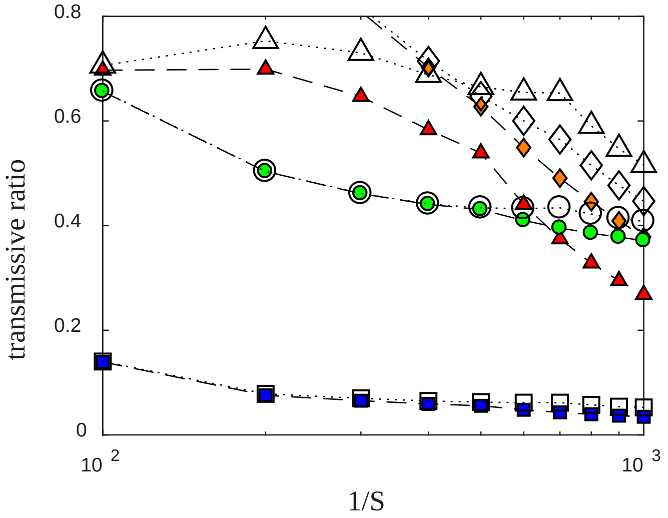

Figure S5. Comparison of transmissive ratio. Coloured symbol: with friction terms. White symbol: without friction terms. Diamond: $\mathcal{T}_{E\phi}$. Triangle: $\mathcal{T}_{Ek}$. Circle: $\mathcal{T}_{Vw}$. Square: $\mathcal{T}_{Mo}$.
